# Thermal tolerance and range expansion of invasive foraminifera under climate changes

**DOI:** 10.1038/s41598-019-40944-5

**Published:** 2019-03-12

**Authors:** Danna Titelboim, Ahuva Almogi-Labin, Barak Herut, Michal Kucera, Sarit Asckenazi-Polivoda, Sigal Abramovich

**Affiliations:** 1 Ben-Gurion University of the Negev, Beer Sheva, Israel; 2 Geological Survey of Israel, Jerusalem, Israel; 3 Israel Oceanographic and Limnological Research, Haifa, Israel; 4 MARUM – Center for Marine Environmental Sciences, University of Bremen, Bremen, Germany; 5 Dead Sea and Arava Science Center, Masada National Park, Mount Masada, Israel

## Abstract

The Eastern Mediterranean is experiencing a large-scale invasion of alien tropical species from the Red Sea. This “Lessepsian invasion” began with the opening of the Suez Canal and is promoted by the ongoing oceanic warming. The environmental differences between the Red Sea and the Mediterranean act as a buffer allowing the invasion of certain species. This provides an opportunity to study the differences in temperature sensitivity between two sibling species of the cosmopolitian foraminifera *Amphistegina*. Both species are very common in the Red Sea. Whilest, only one is a successful invader and the other is absent in the Eastern Mediterranean. Here we show that the two species are different in their temperature sensitivity, which explains their selective invasion into the Mediterranean. These differences demonstrate that in respect to climate change resilient marine species can be distinguished by their ability to compensate for temperature changes by adjusting their physiological performance and by having tolerance to a wider temperature range. Moreover, we demonstrate that selective filtering mechanisms during invasion can prefer species that are more resilient to colder rather than expected warmer temperatures.

## Introduction

The Mediterranean Sea and particularly its eastern basin, is expected to be one of the most affected areas by the ongoing temperature rise, thus biogeographic studies within this “miniature ocean” can present a model system for understanding global patterns in other marine ecosystems^1^. The opening of Suez Canal caused a large-scale, mostly one-way migration (known as Lessepsian invasion) of many organisms from the Red Sea that dramatically and rapidly changed the composition of marine biota in the Eastern Mediterranean^2–6^. This invasion is promoted by the hyper-oligotrophy and rising SST of the Eastern Mediterranean that allows organisms to settle and thrive and often take over the native fauna in many ecological habitats.

**Figure 1:**
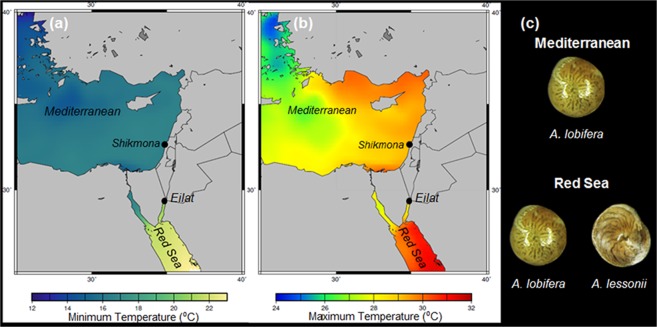
Minimum (**a**) and Maximum (**b**) temperatures in the studied area (temperature data taken from Bio-ORACLE^41,42^). Black dots represent the sampling sites in Shikmona (Eastern Mediterranean) and the Gulf of Aqaba-Eilat. Note the differences in minimum and maximum temperatures between the Red Sea and Mediterranean. In the Eastern Mediterranean, the average range of winter lows-to summer highs is 13 °C–31 °C, compared with 22 °C–28 °C, in the Gulf of Aqaba^8^ (the north-eastern branch of the Red Sea, Fig. 1). (**c**) Digital images of the two studied species *A. lobifera* and *A. lessonii* from the two studied locations.

The Red Sea and the Eastern Mediterranean are similarly saltier than most other oceans, with salinity range between 36–40‰^7^, but different in respect to their sea surface temperatures (SST). In the Eastern Mediterranean the average range of winter to summer SSTs is 13 °C–31 °C, compared with 22 °C–28 °C, in the Gulf of Aqaba^8^ (the north-eastern branch of the Red Sea, Fig. 1). Since Lessepsian invaders are facing major differences in the temperature ranges compared to their oceanic origin (Fig. 1) they can be used to investigate how these changes control biogeographic distribution and invasions.

Such scenarios are well demonstrated by the distribution pattern of the cosmopolitan foraminiferal genus *Amphistegina*. Species of this genus have different physiological traits that governs their species specific distribution patterns^9^. Thus, they present an ideal test case to examine physiological constrains among invading benthic foraminifera species in respect to environmental conditions. Moreover, they are the most abundant foraminifera in carbonate shelves and coral reefs environments^9^, where they play a major role in carbonate production and as ecosystems engineers^10,11^. This genus was also shown to be a good detector for climate change in the geological record^9,12–14^. This highlight its potential to predicted and examine the impacts of climate changes.

The Gulf of Aqaba-Elat, the north-east branch of the Red Sea, is inhabited by two dominant shallow water species of *Amphistegina, A. lobifera* and *A. lessonii*^15,16^. Both are typically found in the same types of habitats. However, only *A. lobifera* successfully invaded the Eastern Mediterranean and is presently found in great numbers along the Israeli Mediterranean coast^8,17,18^. In all surveys of this region, *A. lessonii* was rare or absent^17–21^. Therefore, its absence suggests that unlike the successful incumbency of *A. lobifera*, this species has failed to establish a population in the Eastern Mediterranean. These observations present an ideal test case to investigate the differences in temperature sensitivity between two evolutionary related species as a limiting factor of biogeographic expansion and invasion.

Previous studies have demonstrated that calcification rates can be used as a direct parameter for comparing the temperature sensitivity of different calcifying organisms. This is due to the fact that calcification involves a profound consumption of energy^22–24^. The Alkalinity Anomaly Method is a widely used technique to quantify calcifications rates^24,25^. The efficiency of this parameter was specifically demonstrated on various marine calcifiers including different species of larger benthic foraminifera^8,26–30^.

In this study, we have conducted laboratory culturing experiments that allowed us to compare the temperature sensitivity of three groups: Mediterranean *A. lobifera*, Red Sea *A. lobifera* and Red Sea *A. lessonii* under different temperature conditions.

## Results and Discussion

Ongoing global warming promotes invasion of warm-adapted species into new habitats either by range extension or establishment of alien colonizers. A key to understand future processes of invasions and biogeographical expansion of different species is to study the difference in temperature sensitivity between invasive species and their non-invasive co-occurring siblings, such as in the genus *Amphistegina*.

**Figure 2:**
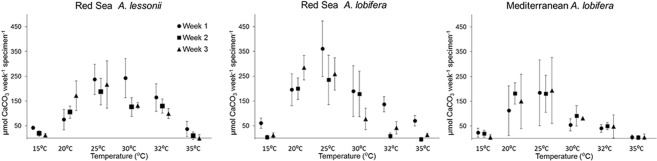
Average calcification rates with respect to temperature of Red Sea *A. lessonii*, Red Sea *A. lobifera* and Mediterranean *A. lobifera*. Error bars are SD. Where error bars are not visible, the uncertainties are smaller than the size of the data point.

Results of this study presented in Fig. 2 and in more detail in supplementary Figure 1 show that the two sibling species of *Amphistegina* from the Red Sea: *A. lobifera* and *A. lessonii*, are clearly different in respect to their temperature sensitivity (also see statistical analyses in Supplementary Tables 2–4). Our approach for examining these differences is to separately compare the response of each group to the temperature treatments. This approach takes into consideration two factors that cause differences in calcification rates between the groups. 1. *A. lobifera* calcify thicker shells than *A. lessonii*^31–33^. This factor is expressed in higher calcification rate of *A. lobifera*. 2. Differences in the reproductive cycle of the two populations of *A. lobifera* from the Red Sea and the Mediterranean^8,19,34^ cause an ontogenetic offset between specimens of the two populations. The effects of these factors are visible in the measured calcification rates of the three groups in the acclimation phase under similar and ideal conditions (Supplementary Table 1a,b).

*Amphistegina lessonii* exhibit a different response to cooler temperatures compared with *A. lobifera*: Minimum calcification rates of *A. lessonii* were recorded during the first week at both 15 °C and 20 °C. An acclimation to 20 °C is indicated by increase calcification rates during the second and third weeks. *Amphistegina lobifera* also exhibit minimum rates at 15 °C throughout the 3 weeks experiment. However, unlike *A. lessonii*, an increase in calcification rates at 20 °C is indicated from week 1.

In the shallow water habitat of the Red Sea, where both species are commonly found, the daily averaged winter temperatures rarely drop below 22 °C^8,35^. In comparison, the daily winter temperatures of the shallow Mediterranean coast of Israel often drop below 15 °C^8,17^ (Fig. 1). Previous studies have already reported that the biogeographic distribution of *Amphistegina* is confined by the winter isotherm of 13.7  °C^9,36–38^. Indeed, our study confirms that the winter temperature minimum of the Eastern Mediterranean coast acts as a physical barrier that prevents the occurrence of *A. lessonii* in this region. Thus, this study not only provides physiological evidences that explain this pattern, but also reveals the different constrains of specific species within this genus.

In respect to higher temperatures, *A. lessonii* exhibit a better tolerance than *A. lobifera*. This observation is based on the onset of significant negative response of *A. lessonii* only at 35 °C. Whilst, *A. lobifera* already exhibit a significant decrease in calcification rate at 32 °C in weeks 2 and 3. The decrease in calcification rate of *A. lessonii* after a sustained time of exposure to elevated temperature (30° and 32°) indicates some sensitivity to higher temperature but clearly not as considerable compared to that of *A. lobifera*. This means that by the time that the winter temperatures in the eastern Mediterranean will be sufficiently high to accommodate *A. lessonii*, the warmer summer temperatures (>32 °C) will likely become a limiting factor for *A. lobifera*.

This prediction agrees with previous observations regarding the presence of *A. lobifera* in a thermally polluted site at the Mediterranean coast of Israel that mimics future temperature rise under natural conditions^17,39^. At the edge of the thermally polluted area where summer temperatures reach up to 33.8 °C, *A. lobifera* is present in low numbers. At the warmest area where summer temperatures rise above 37 °C this species is completely absent.

In respect to climate change, resilient marine species can be distinguished by their better ability to compensate for temperature changes by adjusting their physiological performance^40^, as demonstrated by *A. lessonii* that with time acclimate to 20 °C. Specifically, our results suggest that with a relatively small rise in winter temperatures in the Eastern Mediterranean that will cause the minimum temperature to exceed 20 °C, similar to the Red Sea, the invasion of *A. lessonii* might be enabled. This invasion might be accompanied by similar adaptation as *A. lobifera* that reduced it reproduction from twice to once a year, as observed between the Red Sea and Mediterranean populations.

In order to examine long term effect of invasion on the temperature sensitivity of the invasive population we further evaluated the differences between the invasive and non-invasive populations of *A. lobifera*. Previous study by Schmidt *et al*. (2016) show that both Mediterranean and the Red Sea *A. lobifera* populations responded similarly to elevated temperature based on growth rates and algal performance after 3 weeks exposure. Our results confirm these observations, yet, it implies a different acclimation process between the two populations: The Mediterranean *A. lobifera* seems to be negatively affected at 30 °C already at week 1, whereas, the calcification rates of the Red Sea *A. lobifera* at 30 °C only decrease in week 3 (Fig. 2).

## Conclusion

Physiological plasticity could result in resilience or sensitivity of species specifically since temperature fluctuations will increase under different climate change scenarios and plastic phenotypes should be preferred. Consequently, a wide physiological plasticity will allow species to maintain their fitness throughout a much greater temperature range, whilst the narrow range could lead to limited dispersion ability. Our study demonstrates that in respect to climate changes, resilient marine species can be distinguished by their better ability to compensate for temperature changes by adjusting their physiological performance, as demonstrated by *A. lessonii*: At present, the relatively low winter temperatures of the Eastern Mediterranean produce a physical barrier that impedes the invasion of *A. lessonii*. However, the ability of this species to acclimate to 20 °C and its tolerance to temperatures above 32 °C will allow its invasion and establishment in the Eastern Mediterranean over the following decades. In parallel, the greater sensitivity of Mediterranean *A. lobifera* to warm temperatures will likely disable it to continue thriving in the Eastern Mediterranean. Such distinction is particularly important for a more refined prediction of species response to expected rise in SST.

## Methods

### Specimen collection and handling

Specimens of *Amphistegina* were obtained from pebbles collected from the coast of the Interuniversity Institute of Marine Sciences (IUI), in the Gulf of Aqaba, Eilat, in August and from Shikmona in the northern Mediterranean coast of Israel in July (Fig. 1). In the laboratory, specimens of both species were picked under the binocular and examined for their liveliness: first by detecting typical brownish algal symbionts color and then by motion of the picked specimens from their marked initial location in the petri dishes. To reduce natural ontogenetic variability in calcification rate, only adults (>0.5 mm) were selected for the experiments. Specimens were separated by species, cleaned by brushing and gently transferred into containers with Calcein spiked seawater (~40 µM) and kept in 25 °C. After several days specimens that created new chambers (observed under a fluorescent stereomicroscope) were randomly divided to groups of 20 (Red Sea) or 25 (Mediterranean) specimens and placed in 60 ml airtight Erlenmeyer flasks. For each species and treatment 5–6 replicates were prepared and analyzed. The airtight Erlenmeyer were specifically chosen to prevent evaporation during the experiment. Each Erlenmeyer were filled with natural sea water collected at time of foraminifera sampling.

Erlenmeyer were placed in temperature-controlled water baths (cooled and heated simultaneously) with the exception of those grown in 15 °C that were placed in temperature-controlled incubation chamber (Pol-Eko-Aparatura). All maintaining a constant temperature of at least ± 0.5 °C. During cultivation, samples were kept under white fluorescent light of ~45 µmol photons m^−2^ s^−2^ under 12 hours light 12 h dark cycle. Temperature and light was monitored regularly during the duration of experiments.

### Laboratory manipulative experiments

To determine calcification rates of Red Sea and Mediterranean amphistiginds two sets of experiments were carried out in the culturing laboratory in Ben Gurion University of the Negev, Israel. The following is a description of the methodology used in both experiments: all replicates were initially cultured in 25 °C completing with the calcein stage a total of 10 days for specimens to acclimate in 25 °C prior to the beginning of the experiments. Calcification rate was measured and created a reference for the “calcification activity” between replicates. Moreover, replicates that did not exhibit clear calcification were excluded from the rest of the experiments to avoid any bias to the results. Then, a slow acclimation process was performed until each treatment reached the designated temperature. Each experiment included six temperature treatments: 15 °C, 20 °C, 25 °C, 30 °C, 32 °C and 35 °C broadly representing and exceeding the natural temperature variation in the Gulf of Aqaba and in the Mediterranean coast of Israel. The highest temperature aims to represent future scenario of rising SST.

To evaluate calcification rates of *A. lobifera* and *A. lessonii* under different temperatures we used the alkalinity anomaly method^24^: Briefly, calcification rates were calculated from changes in the total alkalinity (A_T_) of the seawater. These changes were determined by measuring total alkalinity at the end of each incubation period (1 week) and compared to a control sample that contained seawater with no foraminifera. At the end of each incubation period all of the water in the erlenmeyers was replaced with new natural seawater. From the removed water two replicates were taken from each water sample for measurements of the total alkalinity. In case where the differences between the duplicated measurements were larger than 10 µEq L^−1^ a third sample was measured. Accuracy was assessed by analysis of the Scripps Institute of Oceanography reference seawater (batch 154) and an internal standard. Calculated calcification rates were corrected for the volume of seawater, normalized per week and to the number of specimens in each sample (all specimens were of similar size at the beginning of the experiments). Calculated calcification rates of each experiment are presented Supplementary Figure 1 in µmol CaCO_3_ week^−1^ specimen^−1^.

## Supplementary information

Supplementary Figure 1, Supplementary Table 1a, Supplementary Table 1b, Supplementary Table 2a, Supplementary Table 2b, Supplementary Table 3a, Supplementary Table 3b, Supplementary Table 4a, Suppleme

## Data Availability

All data is available in the supplementary materials of the paper.
