# Supplementary material for: Thermal tolerance and range expansion of invasive foraminifera under climate changes
Source: Sci Rep. 2019 Mar 12;9:4198. doi: 10.1038/s41598-019-40944-5 (PMC6414502; doi:10.1038/s41598-019-40944-5)
Supplement: Supplementary file 1 — Supplementary Figure 1, Supplementary Table 1a, Supplementary Table 1b, Supplementary Table 2a, Supplementary Table 2b, Supplementary Table 3a, Supplementary Table 3b, Supplementary Table 4a, Suppleme [file 41598_2019_40944_MOESM1_ESM.pdf]

Thermal tolerance and range expansion of invasive foraminifera under climate changes

Danna Titelboim, Ahuva Almogi-Labin, Barak Herut, Michal Kucera, Sarit Asckenazi-Polivoda, and Sigal Abramovich

Supplementary Figure 1: Calcification rates with respect to temperature of *A. lessonii* from the Red Sea (left) *A. lobifera* from the Red Sea (middle) and *A. lobifera* from the Mediterranean (right) after one (top), two (middle) and three (bottom) weeks. Error bars are 2SD. Where error bars are not visible, the uncertainties are smaller than the size of the data point.

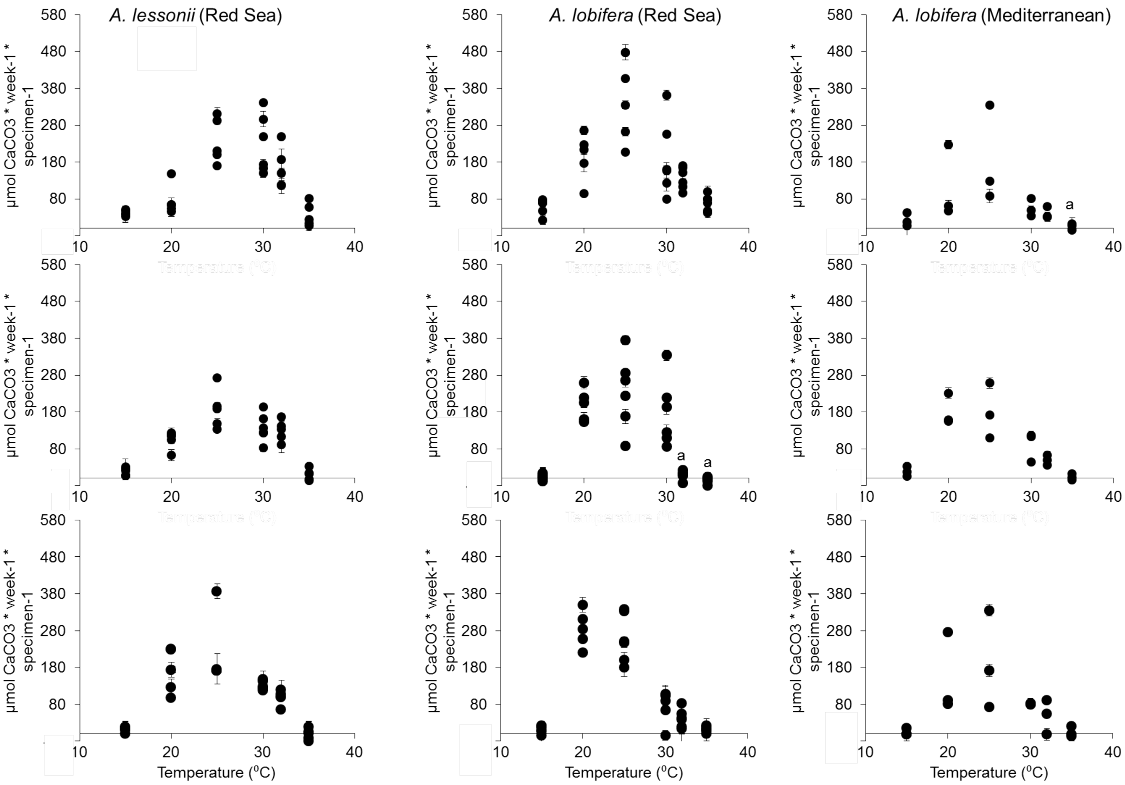

Supplementary Table 1a: One-way ANOVA comparing calcification rates of the three examined groups under similar and ideal conditions.

	SS	Degr. of	MS	F	p
Intercept	2290826	1	2290826	317.8	0.000000
Group	441994	2	220997	30.66	0.000000
Error	619913	86	7208		

Supplementary Table 1b: Tukey HSD post-hoc test demonstrating the differences between calcification rate (µmol $CaCO_3$ week^{-1} specimen^{-1}) of the three groups when cultured under similar and ideal conditions. Stars indicate homogenous groups and thus significant differences between them.

Group	Calcification rate	1	2
Mediterranean *A. lobifera*	96.1	****	
Red Sea *A. lessonii*	133.9	****	
Red Sea *A. lobifera*	258.9		****

Supplementary Table 2a: Results of ANOVA test indicating differences in calcification rate of Red Sea *A. lessonii* between temperature treatments.

	SS	n	MS	F	p
Intercept	366.0812	1	366.0812	11408.01	0.000000
Temp	10.5807	5	2.1161	65.94	0.000000
week	0.4468	2	0.2234	6.96	0.001686
Temp*week	1.1041	10	0.1104	3.44	0.000937
Error	2.4067	75	0.0321		

Supplementary Table 2b: Tukey HSD post-hoc test results indicating significant differences in the response of Red Sea *A. lessonii* to the different temperature treatments in the three weeks of the experiment.

Temp	week	1	2	3	4	5	6	7
35	3	****						
35	2	****	****					
15	3	****	****					
15	2		****	****				
35	1		****	****	****			
15	1		****	****	****	****		
20	1			****	****	****	****	
32	3				****	****	****	****
20	2				****	****	****	****
32	2					****	****	****
30	3					****	****	****
30	2					****	****	****
32	1						****	****
20	3						****	****
25	2						****	****
25	3						****	****
30	1							****
25	1							****

Supplementary Table 3a: Results of ANOVA test indicating differences in calcification rate of Red Sea *A. lobifera* between temperature treatments

	SS	n	MS	F	p
Intercept	410.4727	1	410.4727	10003.87	0.000000
Temp	14.2731	5	2.8546	69.57	0.000000
week	2.7639	2	1.3820	33.68	0.000000
Temp*week	2.6138	10	0.2614	6.37	0.000000
Error	3.5697	87	0.0410		

Supplementary Table 3b: Tukey HSD post-hoc test results indicating significant differences in the response of Red Sea *A. lobifera* to the different temperature treatments in the three weeks of the experiment.

Temp	week	1	2	3	4	5	6	7
35	2	****						
15	2	****	****					
32	2	****	****					
15	3	****	****	****				
35	3	****	****	****				
32	3		****	****	****			
15	1			****	****	****		
30	3			****	****	****	****	
35	1				****	****	****	
32	1				****	****	****	****
30	2					****	****	****
30	1					****	****	****
20	1					****	****	****
20	2						****	****
25	2							****
25	3							****
20	3							****
25	1							****

Supplementary Table 4a: Results of ANOVA test indicating differences in calcification rate of Mediterranean *A. lobifera* between temperature treatments

	SS	n	MS	F	p
Intercept	148.7650	1	148.7650	1138.464	0.000000
Temp	13.1475	5	2.6295	20.123	0.000000
week	0.2751	2	0.1376	1.053	0.359502
Temp*week	0.5002	10	0.0500	0.383	0.946058
Error	4.7042	36	0.1307		

Supplementary Table 4b: Tukey HSD post-hoc test results indicating significant differences in the response of Mediterranean *A. lobifera* to the different temperature treatments in the three weeks of the experiment.

Temp	week	1	2	3
35	3	****		
35	1	****	****	
35	2	****	****	
15	3	****	****	
15	2	****	****	****
15	1	****	****	****
32	3	****	****	****
32	1	****	****	****
32	2	****	****	****
30	1	****	****	****
30	3		****	****
30	2		****	****
20	1		****	****
20	3			****
25	1			****
25	3			****
25	2			****
20	2			****
